# Retraction: NEAT1 Overexpression Indicates a Poor Prognosis and Induces Chemotherapy Resistance via the miR-491-5p/*SOX3* Signaling Pathway in Ovarian Cancer

**DOI:** 10.3389/fgene.2022.861109

**Published:** 2022-01-31

**Authors:** Frontiers Editorial Office

The journal and Chief Editors retract the 29 April 2021 article cited above.

Following publication, concerns were raised regarding the validity of the data in the article. The authors failed to provide the raw data or a satisfactory explanation during the investigation, which was conducted in accordance with Frontiers’ policies. Given the concerns, and the lack of raw data, the editors no longer have confidence in the findings presented in the article.

This retraction was approved by the Chief Editors of Frontiers in Genetics and the Chief Executive Editor of Frontiers. The authors have not responded to any correspondence regarding the retraction.

## Publisher’s Note

All claims expressed in this article are solely those of the authors and do not necessarily represent those of their affiliated organizations, or those of the publisher, the editors and the reviewers. Any product that may be evaluated in this article, or claim that may be made by its manufacturer, is not guaranteed or endorsed by the publisher.

